# Major ECG abnormalities and risk of incident cardiovascular disease in people with HIV compared to people without HIV

**DOI:** 10.1097/QAD.0000000000004547

**Published:** 2026-05-18

**Authors:** Manon C. Vanbellinghen, Iris A.J. van der Wulp, Pieter G. Postema, Jan A. Kors, Maarten F. Schim van der Loeff, Peter Reiss, Marc van der Valk, Anders Boyd

**Affiliations:** a Amsterdam UMC, location University of Amsterdam, Department of Infectious Diseases; b Amsterdam Institute for Immunology and Infectious Diseases; c Amsterdam UMC, location University of Amsterdam, Department of Global Health; d Amsterdam Institute for Global Health and Development; e Amsterdam UMC, location University of Amsterdam, Heart Centre, Department of Cardiology, Amsterdam; f Department of Medical Informatics, Erasmus University Medical Center, Rotterdam; g Public Health Service of Amsterdam, Department of Infectious Diseases; h Stichting hiv monitoring, Amsterdam, The Netherlands.

**Keywords:** cardiovascular diseases, cohort studies, electrocardiography, heart disease risk factors, HIV, prospective studies

## Abstract

**Objectives:**

People with HIV (PWH) have an increased risk of cardiovascular disease (CVD). In the general population, major electrocardiographic (ECG) abnormalities have been associated with increased risk of incident CVD. We compared the role of major ECG abnormalities in CVD between PWH and people without HIV (PWoH).

**Methods:**

PWH and PWoH with ≥1 ECG across six biennial study visits and without prior CVD were included. Transition intensities (TI) between: no major ECG abnormality, major ECG abnormality, and CVD were estimated using a multistate Markov model. Hazard ratios (HRs) were adjusted for age and BMI. *Post hoc* analyses explored whether inflammatory markers influenced the association between HIV-status and transitions.

**Results:**

Overall, 1060 participants were included (540 PWH and 520 PWoH; median age = 52 years, IQR = 48–58). During a median follow-up of 10.8 years interquartile range, (IQR = 6.8–11.0), 69 (12.8%) PWH and 52 (10.0%) PWoH had any major ECG abnormality (*P* = 0.16), while 65 (12%) PWH and 41 (8%) PWoH (*P* = 0.024) had an incident CVD event. There were 55 transitions from no major abnormality to major abnormality (TI = 0.76/100PY, 95% CI = 0.58–1.00) with no difference between PWH and PWoH (adjusted-HR = 1.57, 95% CI = 0.92–2.68). There were 18 transitions from major abnormality to CVD (TI = 3.38/100PY, 95% CI = 1.98–5.75), with a significantly higher TI for PWH (adjusted-HR = 3.24, 95% CI = 1.11–9.55). Adjustment for interleukin-6 attenuated the HR of HIV for this transition by 16%.

**Conclusion:**

PWH with major ECG abnormalities had a significantly higher risk of incident CVD than PWoH, possibly related to systemic inflammation. Given the low probability of developing these abnormalities, routine ECG screening is unlikely to be of benefit.

## Introduction

People with HIV (PWH) are at increased risk of cardiovascular morbidity and mortality compared to the general population, even in the context of sustained viral suppression [1,2]. This elevated risk persists after adjustment for traditional cardiovascular risk factors, suggesting additional HIV-related mechanisms, such as chronic inflammation, immune activation, and possibly side effects of antiretroviral therapy (ART) [3].

An electrocardiogram (ECG) can be used to detect subclinical cardiac abnormalities, such as bundle branch blocks (BBBs), ST-segment and/or T-wave abnormalities and QT prolongation. Major ECG abnormalities have been consistently associated with cardiovascular morbidity and mortality in both the general population [4,5] and to a lesser extent in PWH [6,7].

To date, no study has longitudinally examined the evolution of major ECG abnormalities and their association with incident cardiovascular morbidity and mortality in PWH as compared to people without HIV (PWoH). This study aimed to assess whether transitions between the absence and presence of subclinical major ECG abnormalities, along with their risk of incident cardiovascular disease (CVD), differ between PWH and PWoH without known CVD. Additionally, we aimed to examine whether adjustment for inflammatory markers might attenuate any observed association between HIV status and the transition from no major abnormality to major abnormality, and from major abnormality to CVD. Finally, we evaluated whether HIV-related factors were associated with these transitions among PWH.

## Methods

### Study population

The AGEhIV Cohort Study is a prospective observational study in which participants were enrolled between October 2010 and September 2012. The study evaluates ageing-associated comorbidities in PWH and PWoH, aged 45 years and older. PWH were recruited from the HIV outpatient clinic of the Amsterdam University Medical Centre (Amsterdam, the Netherlands), while participants without HIV were recruited through the Public Health Service of Amsterdam [8].

Standardized screening for ageing-associated comorbidities was conducted at the first study visit and every 2 years thereafter, alongside ECG assessment. Detailed study procedures have been previously published [9]. For PWH, additional information on HIV-related characteristics was obtained from the Dutch HIV Monitoring Foundation (*Stichting hiv monitoring*, SHM) registry [10].

For this analysis, we included all participants who had at least one ECG at one of the six study visits. Participants were excluded if they had a prior diagnosis of CVD (i.e., angina pectoris, cerebrovascular accident, myocardial infarction, peripheral artery disease, or heart failure) or a paced heart rhythm at enrolment.

### ECG measurements

Standard 12-lead ECGs were recorded in supine position with a GE MAC 800 ECG system (GE Healthcare). All ECGs were evaluated for abnormalities by a cardiologist (P.G.P.). ECGs were thereafter processed with the Modular ECG Analysis System (MEANS) program [11], which determines P-wave, QRS and T-wave onsets and offsets for all 12 leads together on one representative averaged beat. All automatically placed on- and offsets were visually assessed and adjusted where needed.

### Outcome variables

The analysis is centred on three mutually exclusive states which participants can occupy at each study visit: absence of major ECG abnormalities, presence of major ECG abnormalities, and incident CVD.

Major ECG abnormalities were classified using Minnesota codes assigned by the MEANS program in accordance with the Minnesota code manual (Supplementary Table 1, Supplemental Digital Content) [12]. ECGs with one or more major abnormalities were compared with the initial assessment provided by the study cardiologist (P.G.P.). If a discrepancy was identified, it was resolved through a secondary review with the same cardiologist.

Incident CVD was defined as any one of the following: angina pectoris, cerebrovascular accident, myocardial infarction, peripheral artery disease, heart failure, cardiovascular death (Supplementary methods, Supplemental Digital Content for definition and adjudication), and pacemaker implantation. These events were validated using clinical records; if validation was not possible, self-reported events were used.

### Covariables

Definitions of covariables are provided in the Supplementary methods, Supplemental Digital Content. Country of origin, sexual orientation, and education level were collected at the first study visit using a standardized questionnaire. Lifestyle variables including smoking, stimulant use, alcohol consumption, and physical activity were assessed and updated at each study visit using questionnaire data. HIV-related variables were defined using data extracted from the SHM registry.

At each study visit, data on medication use, physical measurements (weight, height, waist and hip circumference, blood pressure), and laboratory tests (lipids, glucose) were collected. These were used to assess the presence of hypertension, chronic kidney disease and metabolic syndrome (Supplementary methods, Supplemental Digital Content). The estimated 10-year risk of first onset fatal and nonfatal cardiovascular disease was calculated using the European Society of Cardiology (ESC) Systematic Coronary Risk Evaluation 2 (SCORE2) and SCORE-2OP models [13,14]. Participants with diabetes or with moderate to severe chronic kidney disease were classified according to the appropriate ESC guidelines [15–17].

### Statistical analysis

Baseline was defined as the first study visit with an ECG measurement. Follow-up continued until the date of cardiovascular event, death, study withdrawal, or last ECG measurement, whichever came first.

A time-homogeneous, continuous time multistate Markov model was used to estimate transitions during follow-up between: no major ECG abnormality, major ECG abnormality, and CVD. Once participants developed CVD, they could not transition to any other state (i.e., absorbing state). The Markov model was estimated with maximum likelihood functions, using the ‘msm’ package in R [18]. From this model, the number of transitions per 100 person-years (PY), referred to as transition intensity (TI), was estimated between each of the states. Additionally, 5- and 10-years transition probabilities were estimated; representing the cumulative probability of moving from a starting state to another within a defined time-frame. The primary transitions of interest were from no major ECG abnormalities to major ECG abnormalities, and from major ECG abnormalities to cardiovascular disease. The results focus on these transitions, with other transitions reported where relevant.

To assess the determinants of transitioning between states, covariates were added to the model from which the hazard ratios (HRs) and 95% confidence intervals (CIs) of TIs between levels of covariates were computed. For time-updated covariates with missing data, it was assumed that their values remained unchanged until the next study visit with available data (i.e., last observation carried forward). Goodness-of-fit was evaluated by graphically comparing observed to expected state prevalences over ten years, and using the Pearson-type goodness-of-fit test from the ‘msm’ package in R [18]

In a first univariable model, time-updated HIV status was added as a covariate. PWoH who acquired HIV during follow-up were considered PWH for all subsequent visits. In a second multivariable model, HIV status was added along with determinants associated with any primary transition of interest in univariable analysis (i.e., 95% CI of the HR did not include unity). The determinants considered were age, sex at birth, recent stimulant use, diabetes mellitus, metabolic syndrome, hypertension, current smoking, physical activity, BMI, heavy alcohol use, kidney function (eGFR), and total cholesterol/high-density lipoprotein (TC/HDL) ratio. All covariates were time-updated, except for age and sex at birth. The final multivariable model included age and BMI. Several sensitivity analysis were performed. Participants whose first major ECG abnormality coincided with a CVD event were reclassified as having developed the major abnormality at the preceding visit to assess the effect of event timing on the model. To address residual confounding by traditional cardiovascular risk factors, we added continuous SCORE2/SCORE2-OP to the multivariable model, omitting age as it is included in the risk score. To assess the potential impact of last observation carried forward, the multivariable model was rerun using complete cases only without last observation carried forward. Finally, to account for the possible persistent effects of smoking on CVD risk, participants who reported smoking at the preceding visit but not at the current visit were reclassified as current smokers, introducing an approximate 2-year lag.

Several *post hoc* analyses were conducted. First, the time-updated covariates high-sensitivity C-reactive protein (hs-CRP) and D-dimer, and baseline covariates interleukin (IL)-6, soluble CD14 (sCD14) and soluble CD163 (sCD163) were assessed univariably for associations with the primary transitions of interest. IL-6, sCD14 and sCD163 were only measured at the first study visit, hence only baseline values were used for analysis. Markers significantly associated to one of the primary transitions were separately added to the multivariable model and the percentage change in the HR for HIV-status was calculated for the appropriate transition(s).

Second, while only including PWH, HIV-related covariates of interest were added separately to the multivariable model. These covariates included: time spent with CD4^+^ cell count <200 cells/μl, time since HIV diagnosis, prior AIDS, HIV copy-years, recent abacavir use (within 6 months prior to a study visit), duration of protease inhibitor use, duration of D-drug. All HIV-related covariates were time-updated, except for time since HIV-diagnosis.

Statistical analyses were conducted using R (v4.2.1, Vienna, Austria) and STATA (v17.0, College Station, USA).

## Results

### Description of the study population

All 1148 participants enrolled in the AGEhIV Cohort Study were assessed for eligibility. After exclusion of 57 PWH and 30 PWoH with prior CVD, as well as 1 PWH without any available ECG, 540 PWH and 520 PWoH were included in the analysis. Baseline characteristics and HIV-related characteristics are shown in Table 1 (missing data per variable in Supplementary Table 2, Supplemental Digital Content). At baseline, the median age was 52 years [interquartile range (IQR) = 48–58] in both groups. The majority were male at birth (86% in PWH vs. 85% in PWoH). While most participants originated from the Netherlands, a larger proportion of PWH were of non-European origin compared to PWoH (28% vs. 18%). For PWH, the median time since HIV diagnosis was 12 years (IQR = 6–17), 31% had a history of AIDS-defining illness, and 94% were virologically suppressed.

**Table 1:** Baseline characteristics (October 2010–September 20121), by HIV status.

Characteristic	People with HIV (*n* = 540)	People without HIV (*n* = 520)	*P*-value
Age, years	52 (48–58)	52 (48–58)	0.82
Male sex at birth	465 (86%)	440 (85%)	0.49
Country of origin			<0.001
Netherlands	338 (63%)	384 (74%)	
Europe	49 (9%)	44 (8%)	
Outside of Europe	152 (28%)	91 (18%)	
MSM	373 (73%)	354 (70%)	0.28
Higher education	207 (43%)	277 (57%)	<0.001
Current smoker	171 (33%)	130 (26%)	0.009
Stimulant use	39 (8%)	50 (10%)	0.30
Heavy alcohol use	17 (4%)	30 (6%)	0.05
Physically active	219 (46%)	268 (54%)	0.01
Body mass index, kg/m^2^	24 (22–27)	24 (23–27)	0.09
Total cholesterol, mmol/l	5.33 (4.65–6.02)	5.46 (4.86–6.07)	0.12
LDL cholesterol, mmol/l	3.09 (2.51–3.71)	3.26 (2.65–3.84)	0.005
HDL cholesterol, mmol/l	1.29 (1.03–1.57)	1.38 (1.13–1.67)	<0.001
TC/HDL ratio	4.17 (3.22–5.21)	3.84 (3.04–4.87)	0.003
Triglycerides, mmol/l	1.59 (1.04–2.56)	1.41 (0.95–2.09)	<0.001
hs-CRP ≥2.0 mg/l	202 (38)	128 (25)	<0.001
D-dimer, mg/l	0.21 (0.20–0.33)	0.24 (0.20–0.38)	0.002
IL-6, pg/ml	2.0 (2.0–2.8)	2.0 (2.0–3.3)	0.004
IL-6 ≥10.0 pg/ml	47 (9)	51 (10)	0.53
sCD163, ng/ml	289 (206–418)	244 (178–336)	<0.001
sCD14, ng/ml	1588 (1294–1998)	1353 (1080–1730)	<0.001
Hypertension	214 (40%)	146 (28%)	<0.001
Diabetes	30 (6%)	20 (4%)	0.14
Chronic kidney disease	27 (5%)	11 (2%)	0.012
Metabolic syndrome	144 (27%)	85 (17%)	<0.001
Time since HIV diagnosis, years	12 (6–17)		
HIV-RNA <200 copies/ml	505 (94%)		
HIV copy years, log_10_(copies*y/ml)	5 (4–5)		
CD4 nadir, cells/μl	180 (80–260)		
Time with CD4^+^ cell count <200 cells/μl, years	0.1 (0.0–0.7)		
Current CD4^+^ cell count, cells/μl	576 (437–752)		
Prior AIDS diagnosis	170 (31%)		
Recent abacavir use^b^	74 (14%)		
Duration of protease inhibitor use, years	2 (0–6)		
Ever D-drug use^c^	239 (44%)		
Recent D-drug use^d^	9 (2%)		
Duration of D-drug use, years	0.0 (0.0–3.7)		

*Note.* Data are presented as *n* (%) or median (IQR). Characteristics between groups were compared using the Kruskal–Wallis test, Pearson *χ*^2^ test, or one-way analysis of variance F test, as appropriate.

AIDS, acquired immunodeficiency syndrome; HDL, high-density lipoprotein; HIV, human immunodeficiency virus; hs-CRP, high-sensitivity C-reactive protein; IL-6, interleukin 6; LDL, low-density lipoprotein; MSM, men who have sex with men; sCD, soluble cluster of differentiation; TC, total cholesterol.

^a^ Baseline was defined as visit 2 (*March 2013–June 2014*) for eight participants (2 PWH and 6 PWoH) who had a missing ECG at visit 1.

^b^Use of abacavir within 6 months prior to study visit.

^c^Ever exposure to stavudine, zalcitabine or didanosine.

^d^Use of stavudine, zalcitabine or didanosine within 6 months prior to study visit.

### Description of major ECG abnormalities and incident cardiovascular events

The median follow-up time was 10.9 years (IQR = 5.7–11.2) for PWH and 10.7 (IQR = 9.3–10.9) for PWoH, totalling 9430 PY of follow-up. In total, 4805 ECGs were included in the analysis, with a median of 5 (IQR = 2–6) ECGs for PWH and 6 (IQR = 4–6) for PWoH. ECG parameters at baseline are presented in Supplementary Table 3, Supplemental Digital Content.

At baseline, major ECG abnormalities were observed in 34 (6.3%) PWH and in 25 (4.8%) PWoH (*P* = 0.29). Any major ECG abnormality in at least one study visit was observed in 12.8% (*n* = 69) of PWH and 10.0% of PWoH (*n* = 52) (*P* = 0.16). The most common major abnormalities were major isolated ST-T abnormalities (PWH: *n* = 19 [3.5%] vs. PWoH: *n* = 16 [3.1%]; *P* = 0.69) and any intraventricular block (PWH: *n* = 22 [4.1%] vs. PWoH: *n* = 20 [3.8%]; *P* = 0.85). Major Q waves were more common among PWH than PWoH (*n* = 14 [2.6%] vs. *n* = 5 [1.0%], respectively; *P* = 0.045) (Table 2). During follow-up, 65 (12%) PWH and 41 (8%) PWoH developed incident CVD (*P* = 0.024) (Table 2; cardiovascular deaths are described in Supplementary Table 4, Supplemental Digital Content). The majority of events were validated: 59 (91%) in PWH and 33 (80%) in PWoH.

**Table 2:** Any major electrocardiographic abnormalities and incident cardiovascular events during follow-up (October 2010–August 2023), by HIV status.

ECG abnormality or cardiovascular event	People with HIV (*n* = 540)	People without HIV (*n* = 520)	*P*-value
Major ECG abnormality			
Any major abnormality^a^	69 (12.8%)	52 (10.0%)	0.16
Major Q wave	14 (2.6%)	5 (1.0%)	0.045
Minor Q wave with ST-T abnormality	6 (1.1%)	1 (0.2%)	0.12
Major isolated ST-T abnormality	19 (3.5%)	16 (3.1%)	0.69
Any intraventricular block	22 (4.1%)	20 (3.8%)	0.85
RBBB with LAHB	4 (0.7%)	2 (0.4%)	0.69
Brugada pattern	0 (0%)	0 (0%)	NA
LVH with ST-T abnormality	5 (0.9%)	1 (0.2%)	0.22
Major QT prolongation	3 (0.6%)	3 (0.6%)	0.99
Atrial fibrillation or flutter	9 (1.7%)	9 (1.7%)	0.94
Major AV conduction abnormality	2 (0.4%)	2 (0.4%)	0.99
Other major arrhythmias	0 (0%)	0 (0%)	NA
Supraventricular tachycardia	0 (0%)	0 (0%)	NA
Cardiovascular event			
Any cardiovascular event^a^	65 (12%)	41 (8%)	0.024
Angina pectoris	15 (3%)	12 (2%)	0.63
Cerebrovascular event	14 (3%)	7 (1%)	0.15
Myocardial infarction	17 (3%)	11 (2%)	0.30
Peripheral artery disease	6 (1%)	4 (1%)	0.75
Heart failure	7 (1%)	2 (0%)	0.18
Pacemaker implantation	3 (1%)	3 (1%)	0.64
Cardiovascular death^b^	9 (2%)	4 (1%)	0.23

*Note.* Data are presented as *n* (%). Characteristics between groups were compared using the Pearson *χ*^2^ test, or Fisher's exact test, as appropriate. The eight participants who seroconverted during study follow-up were considered as part of the control group; none had any major abnormality or any cardiovascular event.

AV, atrioventricular; CVD, cardiovascular disease; LAHB, left anterior hemiblock; LVH, left ventricular hypertrophy; NA, not applicable; RBBB, right bundle branch block.

^a^Participants could have had more than one major abnormality or cardiovascular event.

^b^ Cause of death, and available information regarding the circumstances surrounding death are provided in Table 4, Supplemental Digital Content.

### Transition intensities and differences between PWH and PWoH

#### Transitions between no major and major ECG abnormality states

**Fig. 1:**
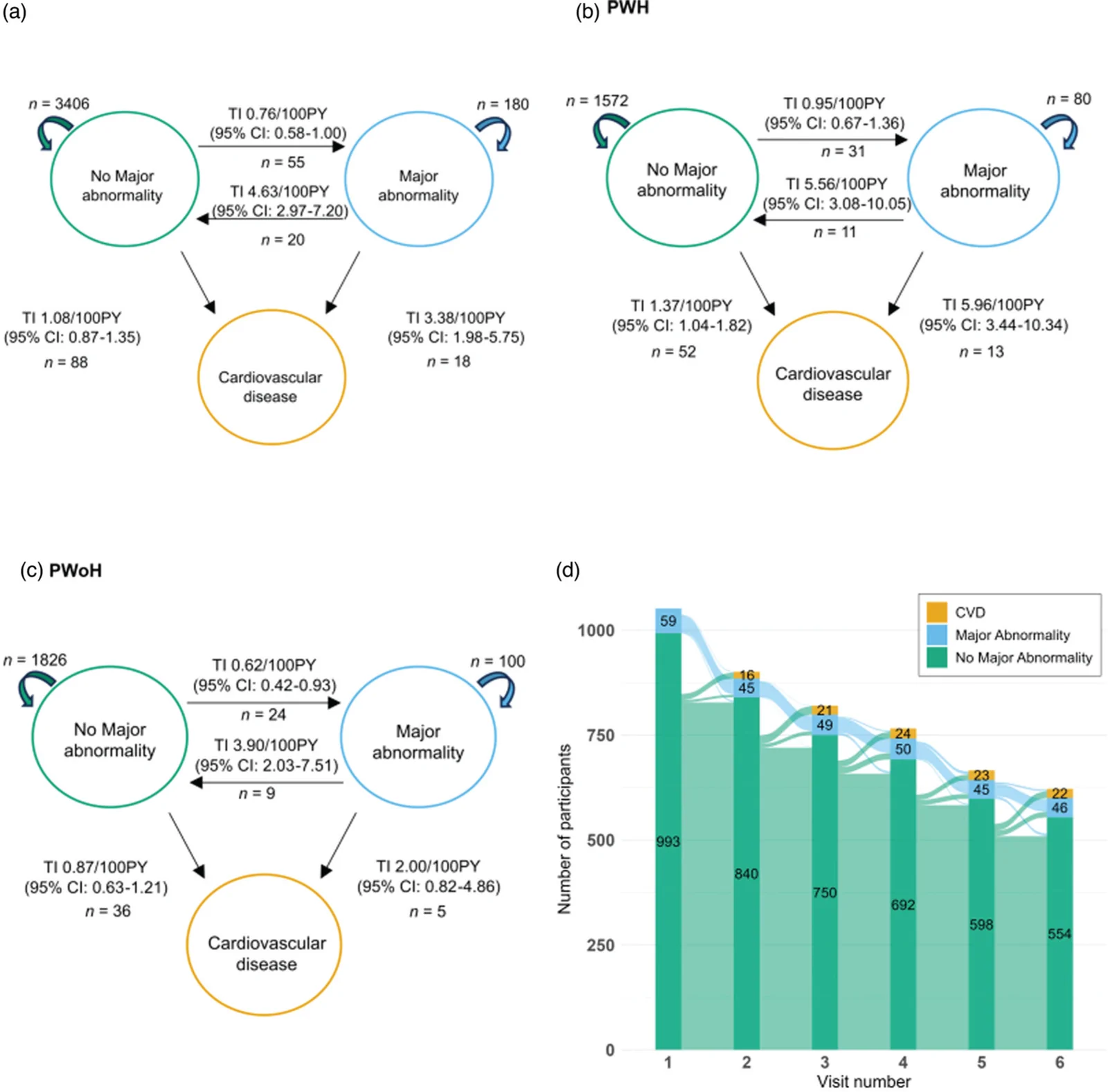
Transitions between ECG states and CVD.

*Note.* Estimated transition intensities per 100 person-years for transitions between no major ECG abnormality, major ECG abnormality and CVD, along with the number of transitions observed over the study period for the entire cohort (a), PWH (b), and PWoH (c). Nonblack arrows represent those participants who remain in the indicated state at each visit. Because eight participants became seropositive for HIV during follow-up, the sum of transitions in the subgroup analyses (b and c) is eight less than in the overall model (a). (d) The number of participants in each state at each study visit and the observed transitions between visits. Participants are counted if they have either an ECG available at the study visit or a registered CVD event. After occurrence of a CVD event, participants are censored as they have entered an absorbing state. CVD, cardiovascular disease; PY, person-years; TI, transition intensity.

During follow-up, there were 55 transitions from no major abnormality to major abnormality, resulting in a transition intensity of 0.76/100PY (95% CI = 0.58–1.00) (Fig. 1a). The number of participants across the states at each study visit, and the transitions between states during follow-up are shown in Fig. 1d. The median time to transition was 6.1 years (IQR = 4.0–8.8), which was similar between PWH (6.2 years, IQR = 4.0–11.0) and PWoH (6.1 years, IQR = 5.9–8.3, *P* = 0.49).

**Fig. 2:**
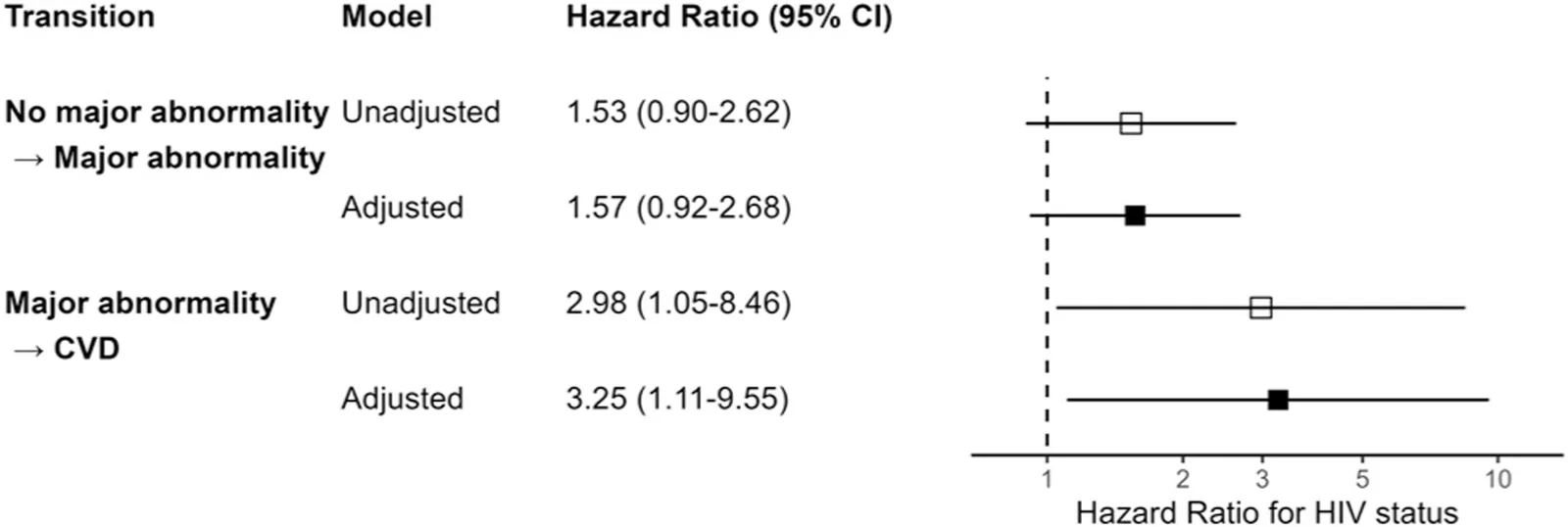
Forest plot of hazard ratios comparing transition intensities of transitions of interest between people with HIV and people without HIV.

*Note.* Forest plot showing the association between HIV status and transitions from no major abnormality to major ECG abnormality or from major ECG abnormality to cardiovascular disease (CVD), as derived from a multistate Markov model. Adjusted hazard ratios are adjusted for age at baseline and time-updated BMI. Graphical depiction of observed and expected values over time for the adjusted model (i.e., to assess goodness-of-fit) is shown in Supplementary Figure 1, Supplemental Digital Content.

**Fig. 3:**
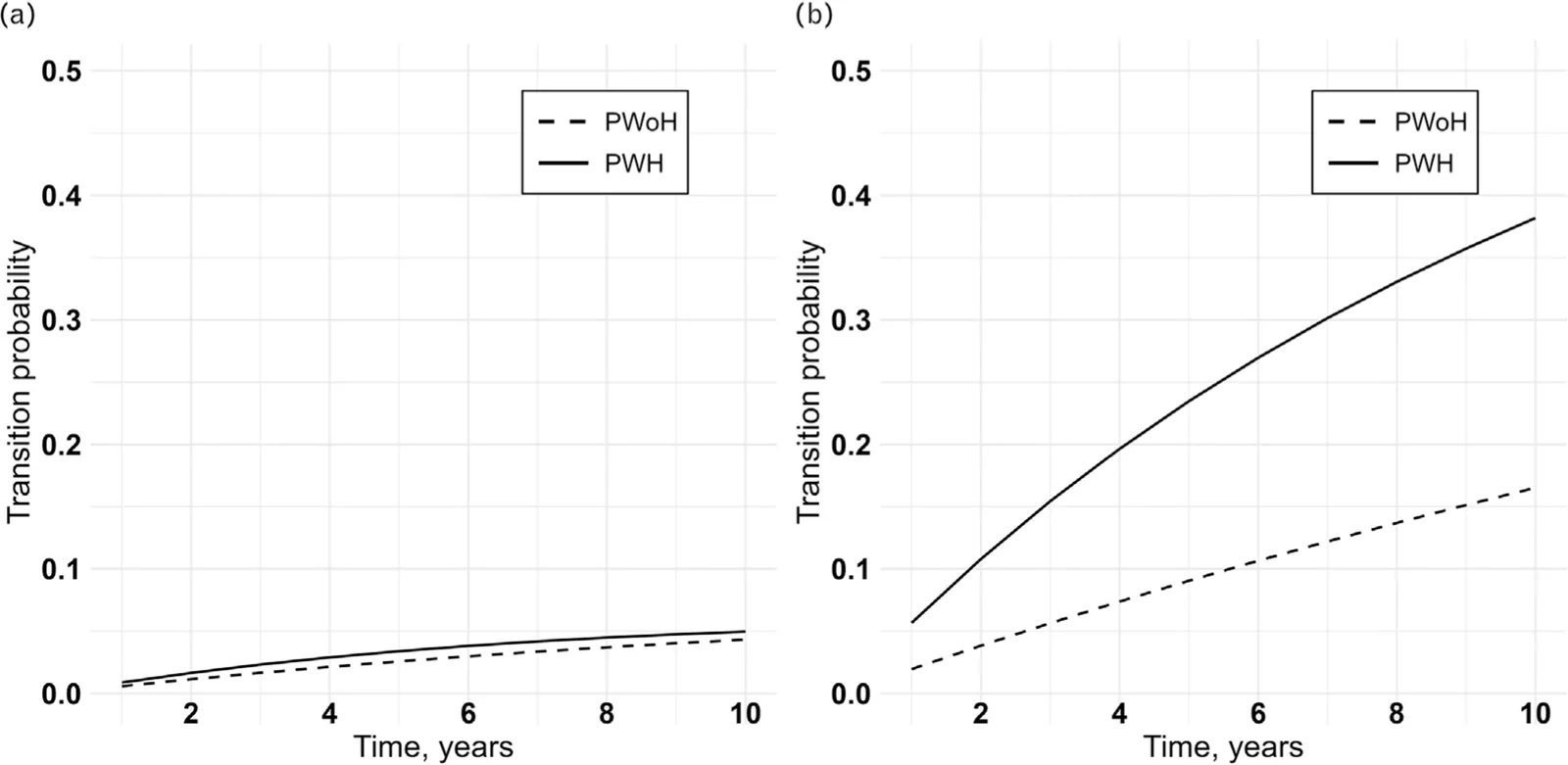
Probability of transitioning from no major ECG abnormality to major ECG abnormality (a) and from major abnormality to cardiovascular disease (b) over time, stratified by HIV status.

*Note.* Modeled probabilities of transitioning between states, as derived from a multistate Markov model, shown over a 10-year period. Transition probabilities represent the cumulative probability of moving from no major ECG abnormality to major ECG abnormality (a) or from major ECG abnormality to CVD (b) over time. Estimates are given separately for PWH and PWoH.

The transition intensity of transitioning from no major abnormality to major abnormality was higher, albeit nonsignificant, in PWH (0.95/100PY, 95% CI = 0.67–1.36, Fig. 1b) than in PWoH (0.62/100PY, 95% CI = 0.42–0.93, Fig. 1c) (unadjusted HR = 1.53, 95% CI = 0.90–2.62). This difference remained nonsignificant after adjusting for age and BMI (adjusted-HR = 1.57, 95% CI = 0.92–2.68) (Fig. 2). The estimated 10-year transition probability was 0.05 (95% CI = 0.03–0.07) for PWH and 0.04 (95% CI = 0.03–0.06) for PWoH (Fig. 3a, Supplementary Table 5, Supplemental Digital Content).

#### Transitions between major ECG abnormality and incident cardiovascular events

Eighteen transitions were observed between major abnormality and CVD, corresponding to a transition intensity of 3.38/100PY (95% CI = 1.98–5.75; Fig. 1a), approximately three times higher than the transition intensity from no major abnormality to CVD (TI-ratio = 3.12; 95% CI = 1.74–5.59). The median transition time was 2.7 years (IQR = 2.0–4.5), and was shorter for PWH (2.5 years, IQR = 2.0–3.4) than PWoH (4.5 years, IQR = 2.1–7.0, *P* = 0.40). The transition intensity of transitioning from major abnormality to CVD was significantly higher in PWH (5.96/100PY, 95% CI = 3.43–10.34, Fig. 1b) than in PWoH (2.00/100PY, 95% CI = 0.82–4.86, Fig. 1c) (unadjusted-HR = 2.98, 95% CI = 1.05–8.46). This difference remained significant after adjusting for age and BMI (adjusted-HR = 3.25, 95% CI = 1.11–9.55) (Fig. 2). The estimated 10-year probability was 0.38 (95% CI = 0.25–0.54) for PWH, and 0.16 (95% CI = 0.08–0.35) for PWoH (Fig. 3b, Supplementary Table 5, Supplemental Digital Content).

Among the eighty-eight transitions from no major abnormality to CVD, seven (4 PWH and 3 PWoH) coincided with a first major ECG abnormality. In the sensitivity analysis, reclassifying these seven individuals as having developed a major ECG abnormality at the preceding visit, the hazard ratios for HIV status were comparable for the transition from no major abnormality to major abnormality (adjusted-HR = 1.59, 95% CI = 0.95–2.66). For the transition from major abnormality to CVD, the hazard ratio remained significant although slightly attenuated (adjusted-HR = 2.51, 95% CI = 1.06–5.91).

In the sensitivity analysis additionally adjusting for SCORE2/SCORE2-OP, hazard ratios were comparable for both the transition from no major abnormality to major abnormality (adjusted-HR = 1.52, 95% CI = 0.89–2.59), and from major abnormality to CVD (adjusted-HR = 3.55, 95% CI = 1.17–10.81).

Supplementary Table 6, Supplemental Digital Content summarizes cardiovascular risk factors, major ECG abnormalities, and CVD events in participants who experienced incident CVD, stratified by ECG-state. Participants who transitioned from a major ECG abnormality to CVD were older and more frequently obese compared with those who transitioned from no major ECG abnormality to CVD. Half of the participants who progressed from a major ECG abnormality to CVD were classified as having a high or very high cardiovascular risk and four (22%) participants had low-density lipoprotein levels within the treatment targets recommended by the ESC. The most frequent abnormalities among participants who experienced a CVD event were intraventricular block (39%) and major isolated ST–T abnormalities (28%).

Univariable hazard ratios for the selected covariates across all transitions are provided in Supplementary Table 7, Supplemental Digital Content. For the transition from no major ECG abnormality to major ECG abnormality, only age was significantly associated in univariable analysis (HR = 1.04, 95% CI = 1.00–1.08). For the transition from major ECG abnormality to CVD, age (HR = 1.07, 95% CI = 1.01–1.14) and BMI (HR = 1.13, 95% CI = 1.04–1.23) were significantly associated. Several traditional cardiovascular risk factors, including diabetes, hypertension, and TC/HDL ratio, were significantly associated with the transition from no major abnormality to CVD; however, none were associated with either primary transition of interest. In the sensitivity analysis using the lagged smoking variable, results were largely consistent with the original smoking variable, except for the transition from no major ECG abnormality to CVD, for which the hazard ratio became significant (HR = 1.61, 95% CI = 1.04–2.49). Univariable and multivariable determinants for the primary transitions of interest are presented in Supplementary Table 8, Supplemental Digital Content. Missingness of time-updated determinants by HIV status, is provided in Supplementary Table 9, Supplemental Digital Content. In the sensitivity analysis using only complete cases, again only age and BMI were included in the multivariable model, and adjusted hazard ratios were unchanged.

### *Post hoc* analyses

#### Possible mediation by inflammatory markers

D-dimer was associated with the transition from no major ECG abnormality to major abnormality (unadjusted-HR = 1.36, 95% CI = 1.12–1.64), its inclusion in the multivariable model increased the HR for HIV-positive status by 3% (Supplementary Figure 2, Supplemental Digital Content). Baseline sCD163 (log_2_-transformed) was significantly associated with the transition from major abnormalities to CVD (unadjusted-HR = 1.93, 95% CI = 1.04–3.58), and its inclusion attenuated the HR of HIV− by 4% (Supplementary Figure 2, Supplemental Digital Content). Baseline IL-6 (log_2_-transformed) was associated with the transition from major abnormalities to CVD (unadjusted-HR = 1.57, 95% CI = 1.25–1.97) and attenuated the HR of HIV by 16% (Supplementary Figure 2, Supplemental Digital Content). Baseline sCD14 and time-updated hs-CRP were not associated with any of the primary transitions of interest.

#### HIV-related determinants of primary transitions of interest in PWH

In analyses restricted to PWH (Supplementary Table 10, Supplemental Digital Content), longer duration of protease inhibitor exposure and recent abacavir use were each associated with increased risk of transitioning to a major ECG abnormality (adjusted-HR = 1.06, 95% CI = 1.01–1.11; and adjusted-HR = 2.38, 95% CI = 1.09–5.18; respectively). Longer protease inhibitor use was also associated with the transition from major ECG to no major ECG abnormality (adjusted-HR = 1.06, 95% CI = 1.00–1.13). Recent abacavir use showed a similar association, although the 95% confidence interval crossed one (adjusted-HR = 3.05, 95% CI = 0.84–11.07). No HIV-related covariates were associated with the probability of transitioning from major ECG abnormality to CVD.

## Discussion

This study examined longitudinal changes in major ECG abnormalities and their association with incident CVD in a prospective cohort of people with and without HIV who did not have CVD at baseline. In the combined cohort, the presence of a major ECG abnormality was associated with an approximately three times higher transition intensity to cardiovascular disease, consistent with previous findings in both the general population and in PWH [6,7,19].

Our study adds to these findings by showing that the cardiovascular risk associated with major ECG abnormalities is more pronounced in PWH, with a modelled 10-year probability of incident CVD at 38% for PWH compared to 16% for PWoH. Notably, the time to incident CVD was also shorter in PWH with a major ECG abnormality, although not statistically significant. However, major ECG abnormalities were uncommon, with the 10-year probability of developing a major ECG abnormality at 5% in PWH and 4% in PWoH. At baseline, the prevalence of any major abnormality was 6.3% in PWH and 4.8% in PWoH and by the end of follow-up, any major ECG abnormality in at least one study visit had been observed in 12.8% and 10.0% of PWH and PWoH, respectively. The baseline prevalence in PWH was notably higher than the 3% reported in the REPRIEVE population, likely reflecting both the inclusion of a lower-risk population in REPRIEVE and differences in the classification of major abnormalities [7].

When examining the separate ECG categories, major Q-wave abnormalities were significantly more prevalent in PWH, consistent with a previous cross-sectional study in PWH and controls [20]. Although not statistically significant when considered separately, other ECG categories associated with ischemia (i.e., minor Q wave with ST-T abnormality and major isolated ST-T abnormality) also appeared somewhat more prevalent in PWH than PWoH when examined together. These findings suggest that PWH may be more likely to have subclinical ECG abnormalities indicative of ischemic changes, which are associated with a higher risk of subsequent CVD, compared to nonischemic ECG abnormalities [5]. Indeed, one study examining incident major abnormalities in PWH found that Q-waves or ST-T abnormalities were the most common *de novo* major ECG abnormality [21]. Soliman *et al.* also found that major isolated ST-T abnormalities were among the more prevalent major ECG abnormalities in PWH, and that this category was strongly associated with incident CVD [6].

The European AIDS Clinical Society (EACS) guidelines recommend an ECG measurement at HIV diagnosis and when initiating certain antiretroviral agents, primarily to detect QTc prolongation and reduce the risk of drug-induced arrhythmias [22]. While routine ECG screening for all PWH may not be justified given the low probability of developing major ECG abnormalities, the incidental finding of a major ECG abnormality should prompt further work-up given the associated elevated CVD risk. A recent substudy from REPRIEVE focussing on ECG abnormalities found that adding ECG findings to traditional risk factors only modestly improved diagnostic performance, hence making routine ECG screening unlikely to substantially improve prediction of future cardiovascular events in PWH [7]. Rather, efforts should focus on improving primary cardiovascular prevention, such as smoking cessation, and weight control. EACS guidelines recommend statin use for all PWH with a CVD risk greater than 5%, and considering statin use in those with lower risk [23]. Notably, in our cohort only approximately 25% of participants who developed CVD had LDL levels within the recommended treatment goals.

Interestingly, in participants with major ECG abnormalities several traditional risk factors, including hypertension, diabetes and TC/HDL ratio, were not significantly associated with the development of CVD, whereas they were in participants without major ECG abnormalities. One possible explanation for this seemingly surprising discrepancy may relate to differences in the type of CVD. In the group with major abnormalities, a substantial proportion had pacemaker implantations due to underlying atrioventricular block. Atrioventricular block is commonly attributed to idiopathic fibrosis of the conduction system, which could partly explain the absence of association with traditional risk factors. It is also possible that chronic inflammation, contributes to the elevated risk of transitioning from major abnormality to CVD [24]. Among the inflammatory markers assessed, IL-6 was significantly associated with the transition from major abnormality to CVD, partially mediating the effect of HIV status. Previous studies have shown that IL-6 is a stronger predictor of CVD compared to hs-CRP and D-dimer in PWH [25,26] and the general population [27]. In PWH on stable ART IL-6 levels have been linked to indicators of atherosclerosis, including carotid intima-media thickness and coronary plaque, [28,29] which may present as ischemic changes on an ECG.

In PWH, both longer exposure to protease inhibitors and recent abacavir use were associated with a higher risk of major ECG abnormalities. However, both factors were also associated with the transition from major abnormality to no major abnormality, although not significantly for abacavir use. These results suggest transient ECG changes in PWH using abacavir or a protease inhibitor, supported by the observation that neither was associated with the transition from major abnormality to CVD.

This study has several limitations. First, the sample size precluded separate analysis of individual major ECG abnormalities, and due to the limited number of incident cardiovascular events, a composite outcome was used. In addition, there were only 18 transitions from major abnormalities to CVD, resulting in greater uncertainty in the estimates, as reflected by the wide confidence interval for the hazard ratio of HIV for this transition. Second, our study population is comprised mainly of men originating from the Netherlands or Europe, limiting the generalizability to other populations. Additionally, as most of our participants had been living with HIV for many years and a notable proportion had experienced severe immunodeficiency, our results might not apply to those diagnosed early and starting treatment with contemporary regimens.

Despite these limitations our study has notable strengths. It was conducted within a well defined population of effectively treated PWH and included a highly comparable control group, allowing us to assess the association between HIV status and major abnormalities. Additionally, the repeated ECG measurements over a substantial follow-up period enabled us to evaluate longitudinal changes in ECG abnormalities and their relationship with incident CVD. Importantly, ECG abnormalities were carefully reviewed and adjudicated, and incident CVD events were validated, strengthening the reliability of our findings.

In conclusion, while HIV status was not associated with the development of major ECG abnormalities, PWH with these abnormalities had a significantly higher risk of incident CVD than PWoH. This excess risk appeared to be partly mediated by IL-6, indicating a role for systemic inflammation. Given the low probability of developing major ECG abnormalities, routine ECG screening is unlikely to be of benefit, though incidental identification of a major abnormality should prompt further cardiovascular work-up.

## Acknowledgements

Author contributions: M.C.V.: Data curation (lead); conceptualization (equal); formal analysis (lead); writing – original draft (lead): writing-review and editing (lead). I.A.J.vdW.: Data curation (equal); project administration (lead); writing – review and editing (supporting). P.G.P.: Conceptualization (supporting); validation (lead); writing – review and editing (supporting). J.A.K.: Software (lead); writing – review and editing (supporting). M.F.S.vdL.: Project administration (equal); writing – review and editing (supporting). P.R.: Conceptualization (equal); supervision (equal); writing – review and editing (equal); funding acquisition (equal). M.vdV.: Conceptualization (equal); funding acquisition (equal); writing-review and editing (equal); supervision (lead). A.B.: Methodology (lead); formal analysis (equal); writing-review and editing (equal); supervision (lead). All the authors have read and approved the text as submitted.

Clinical trial registration: NCT01466582.

Funding: This work was supported by The Netherlands Organization for Health Research and Development [ZonMW, grant number 30002000] and AIDS Fonds [grant number 2009063], and in part by unrestricted research grants from Gilead Sciences; ViiV Healthcare; Janssen Pharmaceuticals N.V.; and Merck Sharp & Dohme Corp. The project was additionally supported by a subsidy from the Dutch Association of HIV-treating Physicians (NVHB). None of these funding bodies had a role in the design or conduct of the study, the analysis and interpretation of the results, the writing of the report, or the decision to publish.

### Conflicts of interest

A.B. has received speaker's fees from Gilead Sciences, Inc. M.F.S.vdL. has received independent scientific grant support from GSK and has served on advisory boards of MSD and Novosanis; all payments were made to his institution. M.vdV. through his institution has received independent scientific grant support and consultancy fees from AbbVie, Gilead Sciences, MSD, and ViiV Healthcare, for which honoraria were all paid to his institution. P.R. through his institution has received independent scientific grant support from Gilead Sciences, Janssen Pharmaceuticals Inc, Merck & Co and ViiV Healthcare, and has in the past served on scientific advisory boards for Gilead Sciences, ViiV Healthcare, and Merck & Co, honoraria for which were all paid to his institution. P.P. has received scientific grant support from the Dutch Heart Foundation. M.C.V., J.K. and I.A.J.vdW. declare no competing interests.

## Supplementary Material

Supplemental Digital Content
