# Supplementary material for: Major ECG abnormalities and risk of incident cardiovascular disease in people with HIV compared to people without HIV
Source: AIDS. 2026 May 18;40(9):1280–90. doi: 10.1097/QAD.0000000000004547 (PMC13566487; doi:10.1097/QAD.0000000000004547)
Supplement: Supplementary file 1 [file aids-40-1280-s001.docx]

**SUPPLEMENTARY DIGITAL CONTENT**

**SUPPLEMENTARY METHODS**

**Definition and adjudication of cardiovascular death**

Cardiovascular deaths were those attributed to underlying cardiovascular disease or natural death of undetermined cause. Deaths were reported either directly to the study team by participant contacts or by the treating HIV physician. In addition, vital status was verified after every study visit for participants lost to follow-up or withdrawn from the study using the Dutch personal records database (Basisregistratie Personen, BPR)^[1]^. Date of death was collected from the BPR or medical records, and cause of death from medical records. The underlying cause of death was classified using the Coding of Death in HIV (CoDe) project methodology^[2]^. Natural deaths of undetermined causes were defined as those in which the direct cause could not be established, but circumstances surrounding the death were known. Deaths with no available information on cause or circumstances were not considered.

1. Government of the Netherlands. **What is the Personal Records Database?** [Internet]. Available from: <https://www.netherlandsworldwide.nl/personal-records-database-brp/what-is-brp> [Accessed 16 April 2024]

2. Kowalska JD, Friis-Møller N, Kirk O, Bannister W, Mocroft A, Sabin C, et al. **The Coding Causes of Death in HIV (CoDe) Project: initial results and evaluation of methodology**. *Epidemiology* 2011; 22(4):516–523.

**Covariable definitions**

Country of origin was categorized according to the Statistics Netherlands (Centraal Bureau voor de Statistiek, CBS) classification: the Netherlands, Europe (excluding the Netherlands), and outside Europe [1]. Men who have sex with men (MSM) were defined as individuals reporting at least two lifetime male-male sexual contacts or those mostly or exclusively sexually attracted to men. Higher education was defined as a bachelor’s degree or higher. Current smoking was defined as having smoked during the last month. Stimulant use was defined as daily to monthly use of MDMA (ecstasy), cocaine, amphetamine (speed), mephedrone (4-MMC), 3-MMC or methylamphetamine (crystal meth) in the preceding six months. Heavy alcohol use was defined as consuming more than four alcoholic drinks per day for men, or two or more drinks for women daily or almost daily over the preceding six months. Physical activity was defined as engaging in moderate physical activity for at least 30 minutes on five or more days per week, or vigorous physical activity for at least 20 minutes on two or more days per week.

1. Statistics Netherlands (CBS). **New classification of population by origin: Replacing classification based on migration background and the concepts western/non-western** [Internet]. Available from: <https://www.cbs.nl/en-gb/longread/statistische-trends/2022/new-classification-of-population-by-origin> [Accessed 16 April 2024].

**Comorbidity definitions**

Hypertension was defined as current use of antihypertensive medication and/or a systolic blood pressure of ≥ 140mmHg or a diastolic blood pressure of ≥ 90mmHg^[1]^. Diabetes was defined as HbA1c ≥ 48 mmol/mol and/or non-fasting blood glucose ≥ 11.1 mmol/L, or fasting blood glucose ≥ 7.0 mmol/L, or current use of antidiabetic medication^[2]^. Chronic kidney disease (CKD) was defined as an estimated glomerular filtration rate (eGFR) calculated with the Chronic Kidney Disease Epidemiology Collaboration **(**CKD-EPI) equation of less than 60 mL/min/1.73 m², and/or a urinary albumin-to-creatinine ratio greater than 30 mg/mmol^[3]^. Metabolic syndrome was defined as the presence of at least three of the following five components: abdominal obesity (waist circumference ≥ 102 cm for men or ≥ 88 cm for women); elevated blood pressure (systolic blood pressure ≥ 130 mmHg and/or diastolic blood pressure ≥ 85 mmHg) or use of antihypertensives; elevated blood glucose (fasting glucose ≥ 5.6 mmol/L or non-fasting glucose ≥ 7.8 mmol/L), or use of antidiabetic medication; low HDL cholesterol (HDL < 1.03 mmol/L for men or < 1.30 mmol/L for women) or use of cholesterol-lowering medication; and elevated triglycerides (≥ 1.7 mmol/L)^[4]^.

1. Mancia G, Kreutz R, Brunström M, Burnier M, Grassi G, Januszewicz A, et al. **2023 ESH Guidelines for the management of arterial hypertension The Task Force for the management of arterial hypertension of the European Society of Hypertension: Endorsed by the International Society of Hypertension (ISH) and the European Renal Association (ERA)**. *J Hypertens* 2023; 41(12):1874–2071.

2. American Diabetes Association Professional Practice Committee. **2. Diagnosis and Classification of Diabetes: Standards of Care in Diabetes—2024**. *Diabetes Care* 2023; 47(Supplement_1):S20–S42.

3. Stevens PE, Levin A. **Evaluation and management of chronic kidney disease: synopsis of the kidney disease: improving global outcomes 2012 clinical practice guideline**. *Ann Intern Med* 2013; 158(11):825–830.

4. Rijksinstituut voor Volksgezondheid en Milieu (RIVM). **Percentage metabool syndroom naar leeftijd en geslacht**. In; 2012

**HIV-related variables definitions**

The following HIV-related data were extracted from the SHM registry: date of first positive HIV test, HIV-RNA copies/ml, CD4+ T-cell counts, acquired immunodeficiency syndrome (AIDS)-defining illnesses and antiretroviral treatments, including their date of commencement and discontinuation. Time since HIV diagnosis was calculated as the years between first positive HIV test and date of study visit. Cumulative HIV copy-years were updated at each study visit by summing the terms of the average HIV-RNA copies/ml divided by time in years between all available measurements ^[1]^. Cumulative time spent with CD4 count <200 cells/μL in years was estimated at each study visit using all available CD4 measurements. Time between measurements was fully counted if both values were below 200, set to zero if both were above, and set to half the interval if the threshold was crossed. AIDS-defining conditions were determined from the US Centers for Disease Control and Prevention classification, and only conditions prior to enrolment were considered. Duration of protease inhibitor or D-drug use (i.e., stavudine, zalcitabine, or didanosine) was calculated as the total number of years a participant used one or more HIV protease inhibitors or D-drugs, respectively. Recent abacavir or D-drug use was defined as any use of these agents within 6 months prior to a study visit.

1. Cole SR, Napravnik S, Mugavero MJ, Lau B, Eron JJ, Jr., Saag MS. **Copy-years viremia as a measure of cumulative human immunodeficiency virus viral burden**. *Am J Epidemiol* 2010; 171(2):198–205.

**SUPPLEMENTARY TABLES**

**Supplementary Table 1**

*Major electrocardiographic abnormalities and corresponding Minnesota Codes*

| Major electrocardiographic abnormality | Minnesota Codes |
| --- | --- |
| Major Q wave abnormalities | 1.1, 1.2 |
| Minor Q wave abnormalities with ST-T abnormalities | 1.3 plus 4.1 or 4.2 or 5.1 or 5.2 |
| Major isolated ST-T abnormalities | 4.1, 4.2, 5.1, 5.2 (without presence of Q waves) |
| Major intraventricular block  Complete or intermittent LBBB  Complete or intermittent RBBB  Nonspecific intraventricular block | 7.1  7.2  7.4 |
| RBBB with LAHB | 7.8 |
| Brugada pattern | 7.9 |
| LVH with ST-T abnormality | 3.1 + 4.1 or 4.2 or 5.1 or 5.2 |
| Major QT prolongation | Major QT prolongation was based on sex-specific Bazett- corrected QT intervals (QTcB ≥ 470 ms for men and QTcB ≥ 480 ms for women). Participants with a QRS duration of ≥120 ms or atrial fibrillation/flutter were not included in the assessment of major QT prolongation. |
| Atrial fibrillation or flutter (continuous or intermittent) | 8.3 |
| Major AV conduction abnormalities^1^  Third-degree AV block  Second-degree AV block  Ventricular preexcitation pattern (WPW) | 6.1  6.2  6.4 |
| Other major arrhythmias | 8.2 |
| Supraventricular tachycardia | 8.4.2  8.4.1 with heart rate ≥ 140 beats per minute |

^1^ Code 6.8, which indicates the presence of an artificial pacemaker and is normally part of the major AV conduction abnormalities, was counted as a cardiovascular outcome and not as a major ECG abnormality.

Abbreviations: AV, atrioventricular; LBBB, left bundle branch block; LAHB, left anterior hemiblock; LVH, left ventricular hypertrophy; ms, millisecond; RBBB, right bundle branch block; WPW, Wolf-Parkinson-White

**Supplementary Table 2**

*Number of missing observations per variable at baseline*

| Variable | People with HIV (n=540) | People without HIV (n=520) |
| --- | --- | --- |
| Age | 0 (0%) | 0 (0%) |
| Sex at birth | 0 (0%) | 0 (0%) |
| Country of origin | 1 (0%) | 1 (0%) |
| MSM | 30 (6%) | 15 (3%) |
| Higher education | 61 (11%) | 31 (6%) |
| Current smoker | 24 (4%) | 13 (3%) |
| Stimulant use | 64 (12%) | 25 (5%) |
| Heavy alcohol use | 62 (11%) | 26 (5%) |
| Physically active | 63 (12%) | 25 (5%) |
| Body mass index | 0 (0%) | 0 (0%) |
| Total cholesterol | 3 (1%) | 24 (5%) |
| LDL cholesterol | 19 (4%) | 29 (6%) |
| HDL cholesterol | 13 (2%) | 24 (5%) |
| TC/HDL ratio | 13 (2%) | 24 (5%) |
| Triglycerides | 7 (1%) | 24 (5%) |
| hs-CRP | 14 (3%) | 0 (0%) |
| D-dimer | 14 (3%) | 6 (1%) |
| IL-6 | 2 (0%) | 3 (1%) |
| sCD163 | 2 (0%) | 6 (1%) |
| sCD14 | 2 (0%) | 3 (1%) |
| Hypertension | 0 (0%) | 0 (0%) |
| Diabetes | 0 (0%) | 0 (0%) |
| CKD | 0 (0%) | 0 (0%) |
| Metabolic syndrome | 1 (0%) | 1 (0%) |
| Time since HIV diagnosis, years | 0 (0%) | … |
| HIV-RNA <200 copies/mL | 3 (1%) | … |
| HIV copy years, log10(copies*y/mL) | 5 (1%) | … |
| CD4 nadir, cells/µL | 0 (0%) | … |
| Time with CD4 count <200 cells/µL, y | 3 (1%) | … |
| Current CD4 count, cells/µL | 0 (0%) | … |
| Prior AIDS diagnosis | 0 (0%) | … |
| Recent abacavir use | 0 (0%) | … |
| Duration of protease inhibitor use, y | 0 (0%) | … |
| Ever D-drug use | 0 (0%) | … |
| Recent D-drug use | 0 (0%) | … |
| Duration of D-drug use, y | 0 (0%) | … |

*Note.* Data are presented as n (%). Abbreviations: AIDS, acquired immunodeficiency syndrome; CKD, chronic kidney disease; HDL, high-density lipoprotein; HIV, human immunodeficiency virus; hs-CRP, high-sensitivity C-reactive protein; IL-6, interleukin 6; LDL, low-density lipoprotein; MSM, men who have sex with men; sCD, soluble cluster of differentiation; TC, total cholesterol.

**Supplementary Table 3**

*Electrocardiographic parameters at baseline, by HIV status*

|  | People with HIV (n=540) | People without HIV (n=520) | *P*-value |
| --- | --- | --- | --- |
| Heart rate, bpm | 65 (58-71) | 63 (58-71) | 0.19 |
| PR, ms | 162 (148-180) | 163 (148-178) | 0.74 |
| QRS, ms | 100 (92-106) | 100 (94-107) | 0.51 |
| QTc, ms | 411 (399-424) | 409 (395-423) | 0.05 |

*Note.* Data are presented as median (IQR). Parameters were compared between groups using the Kruskal-Wallis test One-way analysis of variance F test, as appropriate. Abbreviations: bpm, beats per minute; ms, milliseconds

**Supplementary Table 4**

*Description of 13 participants with cardiovascular or undetermined cause of death*

| HIV status | Age | Sex at birth | Cause of death | Notes |
| --- | --- | --- | --- | --- |
| PWH | 56 | Male | Undetermined | Passed away unexpectedly in sleep |
| PWH | 72 | Male | Undetermined | Found dead at home |
| PWH | 69 | Male | Cardiovascular | Acute myocardial infarction |
| PWH | 76 | Male | Cardiovascular | Acute myocardial infarction |
| PWH | 46 | Male | Cardiovascular | Sudden unexpected death,  presumed cardiovascular cause |
| PWH | 71 | Male | Undetermined | Recent arrhythmia treated with pacemaker implantation |
| PWH | 64 | Male | Cardiovascular | Pulmonary embolism |
| PWH | 73 | Male | Cardiovascular | Acute myocardial infarction |
| PWH | 62 | Male | Cardiovascular | Intestinal ischemia due to thrombosis of both the superior and inferior mesenteric arteries |
| PWoH | 79 | Male | Cardiovascular | Acute myocardial infarction |
| PWoH | 80 | Male | Undetermined | Found dead at home, suspected sudden cardiac arrest |
| PWoH | 62 | Male | Undetermined | Found dead at home; natural death concluded by forensic physician |
| PWoH | 84 | Male | Cardiovascular | Ischemic cerebrovascular accident of the posterior circulation |

Abbreviations: PWH, people with HIV; PWoH people without HIV.

**Supplementary Table 5**

*Estimated 5- and 10-year transition probabilities (95% CI) between no major ECG abnormality, major ECG abnormality and CVD, stratified by HIV status*

|  |  | From no major abnormality | | From major abnormality | |
| --- | --- | --- | --- | --- | --- |
|  |  | To major abnormality | To CVD | To no major abnormality | To CVD |
| PWH | 5 year | 0.03 (0.02-0.05) | 0.07 (0.05-0.09) | 0.20 (0.11-0.31) | 0.23 (0.15-0.36) |
|  | 10 year | 0.05 (0.03-0.07) | 0.14 (0.11-0.18) | 0.29 (0.18-0.45) | 0.38 (0.25-0.54) |
| PWoH | 5 year | 0.03 (0.02-0.04) | 0.04 (0.03-0.06) | 0.16 (0.09-0.28) | 0.09 (0.04-0.19) |
|  | 10 year | 0.04 (0.03-0.06) | 0.09 (0.06-0.12) | 0.27 (0.15-0.44) | 0.16 (0.08-0.35) |

Abbreviations: CVD, cardiovascular disease; PWH, people with HIV; PWoH people without HIV.

**Supplementary Table 6**

*CVD risk factors, major ECG abnormalities, and CVD events in participants with incident CVD*

| Characteristic | Major abnormality  → CVD  (n= 18) | No major abnormality  → CVD  (n=88) | *P*-value |
| --- | --- | --- | --- |
| PWH | 13 (72%) | 52 (59%) | 0.30 |
| Age, y | 61 (58-67) | 56 (50-63) | 0.02 |
| Male sex at birth | 16 (89%) | 80 (91%) | 0.79 |
| Cardiovascular risk category^1^  Low risk  Moderate risk  High risk  Very high risk | 3 (17%)  6 (33%)  7 (39%)  2 (11%) | 22 (25%)  44 (50%)  19 (22%)  3 (3%) | 0.19 |
| Current smoker | 4 (22%) | 33 (38%) | 0.20 |
| Obese (BMI ≥ 30.0 kg/m^2^) | 6 (33%) | 7 (8%) | 0.003 |
| Systolic blood pressure, mmHg | 137 (127-156) | 138 (125-151) | 0.68 |
| Diabetes | 3 (17%) | 9 (10%) | 0.43 |
| HbA1c, mmol/mol | 36 (34-39) | 37 (35-40) | 0.67 |
| CKD | 5 (28%) | 11 (13%) | 0.10 |
| LDL, mmol/L | 2.9 (2.0-3.5) | 3.2 (2.5-4.0) | 0.10 |
| HDL, mmol/L | 1.2 (1.1-1.4) | 1.2 (1.0-1.5) | 0.86 |
| Triglycerides, mmol/L | 1.7 (1.2-3.0) | 1.8 (1.1-3.0) | 0.82 |
| Lipid-lowering drugs | 4 (22%) | 13 (16%) | 0.40 |
| LDL on target^2^ | 4 (22%) | 20 (24%) | 0.96 |
| Major abnormality  Major Q wave  Minor Q wave with ST-T abnormality  Major isolated ST-T abnormality  Any intraventricular block  LVH with ST-T abnormality  Atrial fibrillation or flutter | 3 (17%)  2 (11%)  5 (28%)  7 (39%)  1 (5%)  1 (5%) | --  --  --  --  --  -- |  |
| Cardiovascular event^3^  Angina pectoris  Cerebrovascular event  Myocardial infarction  Peripheral artery disease  Heart failure  Pacemaker implantation  Cardiovascular death | 3 (17%)  2 (11%)  3 (17%)  0 (0%)  3 (17%)  4 (22%)  4 (22%) | 24 (27%)  19 (22%)  25 (28%)  10 (11%)  6 (7%)  2 (2%)  9 (10%) | 0.35  0.31  0.30  0.13  0.17  0.001  0.16 |

*Note.* Data are presented as n (%) or median (IQR). Characteristics between groups were compared using the Pearson χ2 test, or Kruskal-Wallis test, as appropriate. Characteristics are reported at the time of the first recorded major ECG abnormality for participants who transitioned from a major abnormality to CVD, and at the time of the first baseline visit for those who transitioned from no major abnormality to CVD.

One participant in the major abnormality group had two concurrent major ECG abnormalities, so the sum of abnormalities exceeds the total number of individuals in that group.

Data were missing on (major abnormality/ no major abnormality): cardiovascular risk category (0/2), obesity (0/1), systolic blood pressure (0/1), HbA1c (1/3), chronic kidney disease (0/1), LDL (2/5), HDL (0/3), triglycerides (0/3), and lipid-lowering drugs (2/8).

Abbreviations: BMI, body mass index; CKD, chronic kidney disease; HbA1c, haemoglobin A1c; HDL, high-density lipoprotein; LDL, low-density lipoprotein; LVH, left ventricular hypertrophy.

^1^10-year risk category of fatal and non-fatal cardiovascular disease calculated using SCORE2, SCORE2-OP, and SCORE2-Diabetes risk algorithms according to the European Society of Cardiology guidelines ^[1]^.

^2^ Based on treatment goals by cardiovascular risk estimation according to European Society of Cardiology (ECS) guidelines ^[1]^.

^3^ Participants could have had more than one major abnormality or cardiovascular event.

1. Mach F, Koskinas KC, Roeters van Lennep JE, Tokgözoğlu L, Badimon L, Baigent C, et al. **2025 Focused Update of the 2019 ESC/EAS Guidelines for the management of dyslipidaemias: Developed by the task force for the management of dyslipidaemias of the European Society of Cardiology (ESC) and the European Atherosclerosis Society (EAS)**. *Eur Heart J* 2025; 46(42):4359–4378.

**Supplementary Table 7**

*Univariable hazard ratios for determinants of transitions between ECG states and CVD*

|  | No major abnormality  → Major abnormality | Major abnormality  → No major abnormality | No major abnormality  → CVD | Major abnormality  → CVD |
| --- | --- | --- | --- | --- |
| Determinant | HR (95% CI) | HR (95% CI) | HR (95%CI) | HR (95% CI) |
| HIV | 1.54 (0.90-2.62) | 1.42 (0.58-3.44) | 1.58 (1.03-2.44) | 2.98 (1.05-8.46) |
| Age at baseline (per year) | 1.04 (1.00-1.08) | 0.97 (0.91-1.03) | 1.08 (1.05-1.11) | 1.07 (1.01-1.14) |
| Female sex | 0.78 (0.33-1.83) | 1.08 (0.25-4.67) | 0.64 (0.30-1.33) | 1.19 (0.27-5.20) |
| Stimulant use | 1.56 (0.64-3.77) | 5.12 (1.68-15.60) | 0.81 (0.34-1.94) | 1.07 (0.13-9.00) |
| Diabetes | 2.37 (0.94-5.97) | 0.42 (0.06-3.16) | 2.76 (1.36-5.58) | 1.52 (0.43-5.35) |
| Metabolic syndrome | 1.41 (0.78-2.56) | 1.03 (0.37-2.83) | 2.57 (1.66-3.96) | 1.87 (0.71-4.89) |
| Hypertension | 1.31 (0.76-2.28) | 1.35 (0.56-3.26) | 2.12 (1.38-3.24) | 2.19 (0.81-5.91) |
| Current smoking | 0.94 (0.50-1.77) | 2.13 (0.85-5.36) | 1.47 (0.94-2.32) | 0.76 (0.21-2.69) |
| Physically active | 0.86 (0.50-1.47) | 1.23 (0.51-2.96) | 0.62 (0.40-0.99) | 0.98 (0.38-2.51) |
| BMI (per 1 kg/m^2^) | 1.05 (0.98-1.12) | 0.92 (0.81-1.05) | 0.99 (0.93-1.05) | 1.13 (1.04-1.23) |
| Heavy alcohol use | 1.24 (0.38-4.05) | 3.07 (0.71-13.31) | 0.98 (0.36-2.68) | 0.17 (0.00-46.19) |
| eGFR (per 10 mL/min/1.73m^2^) | 0.88 (0.74-1.05) | 1.12 (0.86-1.47) | 0.73 (0.64-0.83) | 0.87 (0.68-1.11) |
| TC/HDL (per 1 unit) | 1.06 (0.87-1.29) | 1.01 (0.73-1.40) | 1.27 (1.12-1.44) | 1.18 (0.87-1.61) |

Abbreviations: BMI, body mass index; CI, confidence interval; CVD, cardiovascular disease; eGFR, estimated glomerular filtration rate; HDL, high-density lipoprotein; HR, hazard ratio; TC, total cholesterol.

**Supplementary Table 8**

*Univariable and multivariable hazard ratios for predictors of transitions from no major ECG abnormality to major ECG abnormality, and from major ECG abnormality to CVD*

|  |  |  | No major abnormality →  Major abnormality | | Major abnormality →  CVD | |
| --- | --- | --- | --- | --- | --- | --- |
|  | *N*  *participants* | *N*  *Observations* | Unadjusted  HR (95% CI) | Adjusted  HR (95% CI) | Unadjusted HR  (95%CI) | Adjusted  HR (95% CI) |
| HIV | 1060 | 5154 | 1.54 (0.90-2.62) | 1.57 (0.92-2.68) | 2.98 (1.05-8.46) | 3.25 (1.11-9.55) |
| Age at baseline (per year) | 1060 | 5154 | 1.04 (1.00-1.08) | 1.04 (1.00-1.07) | 1.07 (1.01-1.14) | 1.04 (0.98-1.11) |
| Female sex | 1060 | 5154 | 0.78 (0.33-1.83) | -- | 1.19 (0.27-5.20) | -- |
| Stimulant use | 1001 | 4953 | 1.56 (0.64-3.77) | -- | 1.07 (0.13-9.00) | -- |
| Diabetes | 1060 | 5154 | 2.37 (0.94-5.97) | -- | 1.52 (0.43-5.35) | -- |
| Metabolic syndrome | 1059 | 5152 | 1.41 (0.78-2.56) | -- | 1.87 (0.71-4.89) | -- |
| Hypertension | 1060 | 5154 | 1.31 (0.76-2.28) | -- | 2.19 (0.81-5.91) | -- |
| Current smoking | 1023 | 5080 | 0.94 (0.50-1.77) | -- | 0.76 (0.21-2.69) | -- |
| Physically active | 998 | 4950 | 0.86 (0.50-1.47) | -- | 0.98 (0.38-2.51) | -- |
| BMI (per 1 kg/m^2^) | 1060 | 5154 | 1.05 (0.98-1.12) | 1.04 (0.98-1.12) | 1.13 (1.04-1.23) | 1.13 (1.02-1.25) |
| Heavy alcohol use | 1022 | 5028 | 1.24 (0.38-4.05) | -- | 0.17 (0.00-46.19) | -- |
| eGFR (per 10 mL/min/1.73m^2^) | 1060 | 5154 | 0.88 (0.74-1.04) | -- | 0.87 (0.68-1.11) | -- |
| TC/HDL ratio (per 1 unit) | 1054 | 5111 | 1.06 (0.87-1.29) | -- | 1.18 (0.87-1.61) | -- |

Abbreviations: BMI, body mass index; CI, confidence interval; CVD, cardiovascular disease; eGFR, estimated glomerular filtration rate; HDL, high-density lipoprotein; HR, hazard ratio; TC, total cholesterol.

**Supplementary Table 9**

*Missing observations per time-updated determinant at visits with an ECG measurement, stratified by HIV status*

| Variable | Visits in PWH (n=2243) | Visits in PWoH (n=2479) | *P*-value |
| --- | --- | --- | --- |
| Current smoker | 24 (1%) | 13 (0.5%) | 0.034 |
| Stimulant use | 232 (10%) | 161 (6%) | 0.000 |
| Heavy alcohol use | 63 (3%) | 26 (1%) | 0.000 |
| Physically active | 220 (10%) | 159 (6%) | 0.000 |
| Body mass index | 3 (0%) | 0 (0%) | 0.069 |
| TC/HDL ratio | 14 (1%) | 41 (2%) | 0.001 |
| Hypertension | 0 (0%) | 0 (0%) | NA |
| Diabetes | 0 (0%) | 0 (0%) | NA |
| eGFR | 1 (0%) | 4 (0%) | 0.22 |
| Metabolic syndrome | 1 (0%) | 4 (0%) | 0.22 |
| Cardiovascular risk score | 33 (1%) | 53 (2%) | 0.087 |

*Note.* Data are presented as n (%). Visits where participants entered an absorbing state were not considered, as covariate values at such visits do not influence the model. Abbreviations: CVD, cardiovascular disease; eGFR, estimated glomerular filtration rate; HDL, high-density lipoprotein; TC, total cholesterol.

**Supplementary Table 10**

*Hazard ratios for HIV-related predictors of transitions between ECG states and CVD in PWH, adjusted for age at baseline and time-updated BMI*

|  | *N^1^ participants* | *N*  *Observations* | No major abnormality → Major abnormality | Major abnormality  → No major abnormality | No major abnormality  → CVD | Major abnormality  → CVD |
| --- | --- | --- | --- | --- | --- | --- |
|  |  |  | HR (95% CI) | HR (95% CI) | HR (95%CI) | HR (95% CI) |
| Time with CD4 < 200 cells/µL (per year) | 543 | 2504 | 1.01 (0.80-1.26) | 0.80 (0.35-1.83) | 1.11 (0.97-1.26) | 1.17 (0.96-1.43) |
| Duration HIV diagnosis (per year) | 546 | 2512 | 0.98 (0.93-1.04) | 0.96 (0.85-1.08) | 0.99 (0.95-1.03) | 1.02 (0.92-1.13) |
| Prior AIDS | 546 | 2512 | 1.09 (0.51-2.35) | 0.76 (0.20-2.88) | 1.39 (0.78-2.46) | 1.68 (0.52-5.40) |
| HIV copy years (per log10(c*y/mL)) | 542 | 2500 | 0.91 (0.59-1.40) | 1.44 (0.56-3.70) | 0.85 (0.61-1.19) | 0.84 (0.36-1.99) |
| Recent abacavir use | 546 | 2512 | **2.38 (1.09-5.18)** | 3.05 (0.84-11.07) | 1.37 (0.70-2.68) | 1.44 (0.40-5.24) |
| PI exposure (per year) | 546 | 2512 | **1.06 (1.01-1.11)** | **1.06 (1.00-1.13)** | 1.04 (1.00-1.07) | 1.02 (0.92-1.12) |
| D-drugs exposure (per year) | 546 | 2512 | 1.01 (0.93-1.11) | 1.03 (0.84-1.25) | 1.01 (0.94-1.08) | 1.02 (0.86-1.21) |

*Note*. Hazard ratios are shown in bold when they do not contain unity (1). Estimates are adjusted for age at baseline and time-updated BMI.

Abbreviations: c, copies; CVD, cardiovascular disease.

^1^ Number of participants in the model. This number may exceed the 540 PWH included at baseline, as 8 PWH seroconverted during follow-up and were subsequently included in the Markov model as PWH.

**SUPPLEMENTARY FIGURES**

**Supplementary Figure 1**

*Goodness-of-fit of the multivariable multi-state model*

*Note*. Modelled and observed prevalences of no major ECG abnormality, major ECG abnormality, and cardiovascular disease (CVD) over 10 years of follow-up. Modelled prevalences are derived from the multivariable multi-state model adjusted for age at baseline and time-updated BMI. Goodness of fit assessed using the Pearson-type goodness-of-fit test was also adequate (p=0.20).

**Supplementary Figure 2**

*Forest plot of hazard ratios comparing transition intensities of transitions of interest between people with HIV and people without HIV after additional adjustment for inflammatory markers*

*Note.* Forest plot showing the showing the association between HIV status and transitions from no major abnormality to major ECG abnormality or from major ECG abnormality to cardiovascular disease (CVD). Adjusted hazard ratios are adjusted for age at baseline and time-updated BMI. D-dimer was included as a time-updated covariate, sCD163 and IL-6 were measured at baseline. sCD163 and IL-6 were log₂-transformed to avoid numerical overflow.
